# miRNAs as potential biomarkers for the progression of gastric cancer inhibit CREBZF and regulate migration of gastric adenocarcinoma cells

**DOI:** 10.7150/ijms.42654

**Published:** 2020-02-24

**Authors:** Yu Jin Kim, Seongtae Jeong, Woon Yong Jung, Jung-Won Choi, Ki-Chul Hwang, Sang Woo Kim, Yong Chan Lee

**Affiliations:** 1 Division of Gastroenterology, Department of Internal Medicine, Kangnam Sacred-Heart Hospital, Hallym University Medical Center, Hallym University College of Medicine, Seoul Korea.; 2 Yonsei University College of Medicine, 50-Yonsei-ro, Seodaemun-gu, Seoul, Republic of Korea.; 3 Institute for Bio-Medical Convergence, College of Medicine, Catholic Kwandong University, Gangneung-si, Gangwon-do 210-701, Republic of Korea.; 4 Department of Pathology, Hanyang University Guri Hospital, Hanyang University College of Medicine, Kyoungchun-ro 153, Guri-si, Republic of Korea.; 5 Catholic Kwandong University, International St. Mary's Hospital, Incheon Metropolitan City, 22711, Republic of Korea.; 6 Division of Gastroenterology, Department of Internal Medicine, Yonsei University College of Medicine, 50-Yonsei-ro, Seodaemun-gu, Seoul, Republic of Korea.

**Keywords:** microRNA, CREBZF, gastric adenocarcinoma, hsa-miR-421, hsa-miR-29b-1-5p.

## Abstract

In our previous study, we identified three miRNAs (hsa-miR-421, hsa-miR-29b-1-5p, and hsa-miR-27b-5p) with two mRNAs (FBXO11 and CREBZF) that might play an important role in the development of gastric adenocarcinoma (GAC) from premalignant adenomas. However, the expression and function of these miRNAs have not been not well characterized. We investigated the roles of CREBZF and miRNAs as potential biomarkers for the progression of gastric cancer (GC) in low-/high-grade dysplasia and early gastric cancer patients using immunohistochemical staining and miRNA *in situ* hybridization. Considering that targets can modulate in GC, we analyzed the CREBZF expression in gastric cancer cell lines by RT-PCR and western blot analysis. We observed lower expression of CREBZF with increasing miRNAs in the MKN-74 gastric cancer cells compared to that in SNU-NCC-19. Next, the role of CREBZF in MKN-74 gastric cancer cells was investigated via cell viability and migration assays by miRNA/anti-miRNA modulation. Furthermore, we found that hsa-miR-421/hsa-miR-29b-1-5p target CREBZF and might play an important role in the migration of MKN-74 cells. This study suggests that increased CREBZF by hsa-miR-421/hsa-miR-29b-1-5p inhibition may be important to prevent the progression of gastric cancer in its early stage.

## Introduction

Gastric cancer (GC) is one of the most common cancer types and is a global health problem 1,2. Although medical treatments have improved, the molecular mechanism underlying oncogenes and tumor suppressors is still not fully understood. Therefore, it is necessary to investigate the etiology of GC and develop more effective prevention strategies and treatments for GC patients.

CREBZF, also known as Zhangfei, is a strong transcriptional activator of HCFC1, which suppresses the HSV protein in cells infected with the virus in an HCFC1-dependent manner 3,4. Moreover, CREBZF cooperates synergistically with HEY1 to enhance p53 transcriptional activity and may participate in the modulation of p53 tumor suppressor function. However, there have been few reports regarding the tumor suppressive role of CREBZF and we were the first to describe the important role of CREBZF and microRNAs (miRNA) in GC development from premalignant adenomas.

Aberrant expression of miRNAs has been observed in various kinds of cancers, including gastric cancer. miRNAs are small non-coding RNA molecules (19-23 nucleotides long) and are known to be involved in tumor development/progression 5,6. miRNAs are considered potential risk factors and are associated with an increased risk of cancer. They also play a role in the formation and progression of GC but little is known about their role in premalignant adenomas. In a previous study, we reported that three miRNAs (hsa-miR-421, hsa-miR-29b-1-5p, and hsa-miR-27b-5p) in conjunction with two mRNAs (FBXO11 and CREBZF) might play an important role in gastric adenocarcinoma (GC) development from premalignant adenomas 7. Changes in the expression patterns of miRNAs may be important and useful in understanding the sequence of progression of gastric adenoma-carcinoma, but precancerous tissues (such as dysplasia/adenoma) have been investigated less often than cancerous tissues 8-10. The molecular characteristics of adenoma, especially of biopsy specimens, have not been fully elucidated.

We postulated that CREBZF and the miRNAs were potentially novel tumor biomarker for the progression of GC in low-/high-grade dysplasia and early gastric cancer patients. Considering that targets can modulate in GC, we investigated the functions of CREBZF with miRNAs in GC cells. We confirmed the negative regulation of CREBZF by miRNAs (hsa-miR-421 and hsa-miR-29b-1-5p) and also showed that loss of miRNA expression leads to an increase CREBZF expression in GC, which in turn decreases cell migration in GC.

## Materials and Methods

### Clinical samples

Human tissue samples were obtained from 12 patients who underwent endoscopic submucosal dissection (ESD) at International St. Mary's Hospital of the Catholic Kwandong University, the donors' basic information with tumor stages is shown in Table 1. The study protocol was approved by the ethics review committee of the Institutional Review Board (IRB no IS17TASI0076), College of Medicine, Catholic Kwandong University. Written informed consent was obtained from individual patients for the use of their tissue samples.

### Cell culture

Two gastric adenocarcinoma cell lines (SNU-NCC-19 and MKN-74) were obtained from the Korean Cell Line Bank (Korea) and maintained in RPMI 1640 medium supplemented with 10% heat inactivated fetal bovine serum, 100 U/ml penicillin, and 0.1 mg/ml streptomycin at 37℃ in a humidified incubator with 5% CO_2_.

### miRNA extraction and quantitative RT-PCR

Total miRNAs were isolated from MKN-74 or SNU-NCC-19 cells using the Quick-RNA miniprep kit (ZYMO research, Orange, CA, USA) according to manufacturer's protocol. A BioFuture MD2000 spectrophotometer was used to measure the concentration of the extracted miRNAs. Extracted miRNA (10 ng) was converted to cDNA using the Taqman advanced miRNA cDNA synthesis kit (Applied Biosystems, Foster city, CA, USA) following the manufacturer's protocol. miRNA expression was analyzed by Quantitative-reverse transcription-PCR using Taqman Fast Advanced Master Mix (Applied Biosystems) on a StepOnePlus Real-Time PCR system. Briefly, 5 μl cDNA was added in 20 μl reaction mixtures comprised of 10 μl TaqMan fast advanced Master Mix (Applied Biosystems), 1 μl Taqman advanced miRNA assay (20 x) primer (Applied Biosystems) and 4 μl Nuclease free water. The quantitative RT-PCR was performed under the following conditions: enzyme activation step at 95℃ for 20 sec, 40 cycles of denaturation at 95℃ for 1 sec and final annealing-extension at 60℃ for 20 sec. For normalization, U6 control transcripts were used and the relative amounts were quantified.

### Real-Time RT-PCR

The level of each gene transcript was quantitatively determined using a StepOnePlus Real-Time PCR System (Applied Biosystems, Foster City, CA, USA). Total RNA was isolated from rat hearts using TRIzol reagent (Invitrogen), and reverse-transcription was performed using a Maxime RT Premix kit (iNtRON Biotechnology, Seongnam, Korea). A SYBR Green Dye system [SYBR Premix Ex Taq (Tli RNase Plus)] with an ROX reference dye (TaKaRa Bio Inc., Foster City, CA, USA) was used to perform real-time RT-PCR. The level of each gene transcript (FBXO11 and CREBZF) was normalized to GAPDH transcript levels, and relative changes in gene expression were quantified using the ∆∆CT method. Table 2 lists all the primers.

### Immunoblot analysis

Cells were lysed with RIPA buffer (Thermo Fisher Scientific) containing 1% phosphatase inhibitor and 1% protease inhibitor (Roche). Protein concentrations were determined using the BCA Protein Assay kit (Thermo Fisher Scientific) and 10 µg protein was diluted in sample buffer (50 mM Tris of pH 6.8, 2% SDS, 10% glycerol, 0.1% bromophenol blue, and 5% β-mercaptoethanol) and heated for 5 min at 99°C. After proteins were separated by SDS-polyacrylamide gel electrophoresis (PAGE) and transferred to a polyvinylidene difluoride (PVDF; Millipore) membrane. The membrane was blocked with 5% skim milk in Tris-buffered saline/0.1% Tween 20 buffer (TBS-T) for 30 min at room temperature and incubated with a 1:1000 dilution of primary antibodies (FBXO11 (Santacruz) and CREBZF (LSBio)) in TBS-T buffer containing 5% bovine serum albumin (AMRESCO, Solon, Ohio, USA) and 0.02% sodium azide (Sigma-Aldrich) overnight at 4°C. After five washes, the membrane was incubated for 1 h with horseradish peroxidase (HRP)-conjugated anti-mouse IgG or anti-rabbit IgG (1:2000, Santa Cruz Biotechnology) in blocking buffer and then washed five times. They were visualized using an enhanced chemiluminescence (ECL, Western Blotting Detection kit, GE Healthcare) system, and the band intensities were quantified using ImageJ software.

### Transfection of miRNA and miRNA inhibitor

We purchased hsa-miR negative, hsa-miR-421, hsa-miR-29b-1-5p, hsa-miR-421 inhibitor, hsa-miR-29b-1-5p inhibitor from Genolution (Genolution Pharmaceuticals, Seoul, Korea). Transfection of miRNA was performed using TransIT-X2 system (Mirus Bio LLC, Madison, WI, USA) for 12 h. SNU-NCC-19 and MKN-74 cells were transfected with a final concentration of 50 nM according to the manufacturer's instructions. Anti-hsa-miR-421 and anti-hsa-miR-29b-1-5p at 50 nM were used to inhibit the expression of endogenous hsa-miR-421 and hsa-miR-29b-1-5p, respectively.

### Luciferase assay

Recombinant CREBZF vector containing CREBZF 3'-UTR was used purchased from GeneCopoeia. The recombinant negative control vector did not contain CREBZF 3'-UTR. MKN-74 was seeded into 96-well plates (3 × 10^4^ cells/well) and transfected using TransIT-X2 system (MIRUS Bio, Madison, WI, USA) as per manufacturer's instructions. Briefly, 50 μM of miR-421, miR-29-1 or negative control mimics were co-transfected with the recombinant CREBZF vector (100 ng) or a negative control vector (100 ng) into the cells. After 24 h, culture medium was changed and the activation of Gaussia luciferase (GLuc) and secreted alkaline phosphatase (SEAP) was determined every 24 h for 48 h using secrete-Pair Dual Luminescence Assay Kit (GeneCopoeia Biotechnolohy, Rockville, MD, USA) following the manufacturer's instructions. Luminescence activity was measured by a microplate reader. Signal normalization was calculated using SEAP signal as an internal standard control to eliminate the impact of transfection efficiency variation (GLuc/SEAP ratio).

### Cell migration assay

The cell migration assays were performed using a 24 well culture plate with silicone culture insert (Ibidi, LLC, Munchen, Germany) which have two individual wells for cell seeding. Briefly, 5 × 10^5^ MKN-74 cells/ml were plated in silicone insert wells in a 24 well culture plate. The silicone inserts restricted cell seeding to the outer regions of the wells. Space between cells was approximately 500 μm. The next day, seeded cells were transfected with 50 nM of miRNA mimics and inhibitors (miR-421 or miR-29-1-5p). After transfection for 24 h, the inserts were removed from the culture plates. The cells were washed with PBS to remove debris and were filled with medium containing 5 % FBS. Photographs of the spaces were taken at 0 h and every 24 h for 3 days with an inverted phase contrast microscope (40 ×).

### Immunohistochemistry (IHC)

Formaldehyde-fixed paraffin-embedded tissues (4 μm sections) were used for IHC staining performed on BenchMark XT (Roche) with optiView system. Counterstaining was performed with hematoxylin solution. Sections incubated with anti-CREBZF (LSBio, Inc., Seattle, WA, USA) at a dilution of 1:200 for 32 min. Standard-technique for BenchMark XT with optiView system was employed.

### miRNA *in situ* hybridization (ISH)

miRNA ISH was carried out on formalin-fixed and paraffin embedded (FFPE) tissue sections according to the kit manufacturer's instructions (miRCURY LNA™ microRNA ISH Optimization Kit; Exiqon Inc., Vedbaek, Denmark). Briefly, the sections were deparaffinized in xylene and rehydrated with graded ethanol with final wash in PBS. The sections were then incubated with Proteinase-K, and hybridized with the miR-421, miR-29-1-5p double-digoxigenin (DIG)-labeled LNA™ probe. A specific anti-DIG antibody directly conjugated with alkaline phosphatase (AP) was applied, and then the sections were incubated the slide in KTBT buffer. The slides were counterstained with Nuclear Red (VECTOR Laboratories Inc., CA, USA).

### Statistical analysis

All experimental results were compared using one-way analysis of variance (ANOVA) in the Statistical Package of Social Science (SPSS, version 17) program. The data were expressed as the mean ± SEM. A protected least-significant difference (LSD) test, which is a method for analyzing multiple comparisons that consist of single-step procedures in one-way ANOVA, was used to identify significant differences between means (*p* < 0.05).

## Results

### hsa-miR-421 and hsa-miR-29b-1-5p expression negatively correlates with CREBZF expression in GC cells

**Figure 1:**
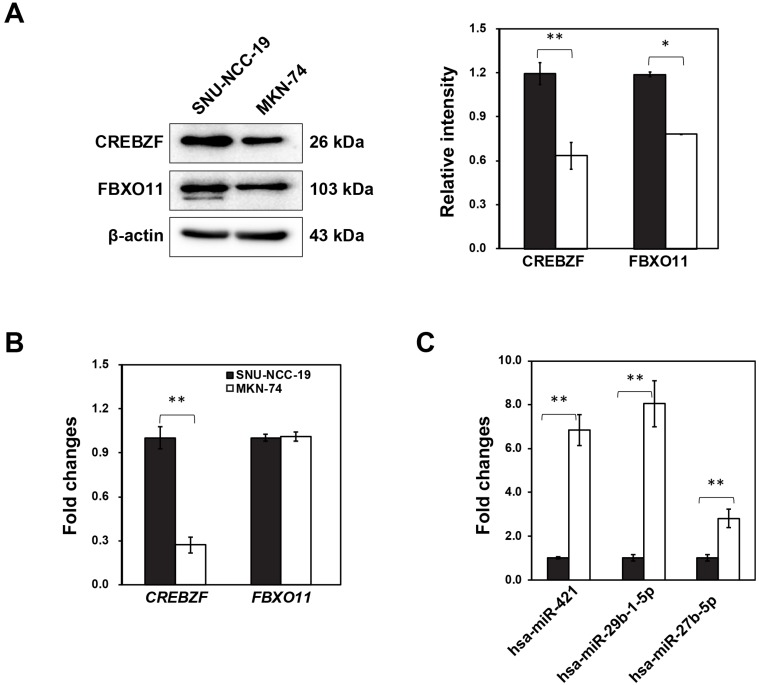
** Differential regulation of potential biomarkers (FBXO11 and CREBZF) and miRNAs (hsa-miR-421, hsa-miR-29b-1-5p, and hsa-miR-27b-5p) in two different gastric adenocarcinoma cell lines (SNU-NCC-19 and MKN-74).** (A) qRT-PCR, (B) Western blot analysis, and (C) Expression level of hsa-miR-421, hsa-miR-29b-1-5p, and hsa-miR-27b-5p. All values are representative of three independent experiments with the S.D. indicated by error bars. Significant differences between the normal and the cancer group were determined via ANOVA, with p values indicated as *p<0.05 and **p<0.01.

Our previous study indicated that three microRNAs in conjunction with two mRNAs might play an important role in the development of GC from premalignant adenoma through network-based visual analysis (miRNet: https://www.mirnet.ca) 7. Considering that targets can modulate in GC, we investigated the differential expression of two targets (*CREBZF* and *FBXO11*) with three miRNAs (hsa-miR-421, hsa-miR-29b-1-5p, hsa-miR-27b-5p) using two different cancer cell lines (SNU-NCC-9 and MKN74). Furthermore, we detected *CREBZF* and *FBXO11* at both mRNA and protein level by real-time PCR and western blot analysis. Expression levels of CREBZF and FBXO11 proteins in MKN74 cells were significantly down-regulated compared to SNU-NCC-9 (Fig. 1A). However, mRNA expression of *FBXO11* was not significantly different between two cell lines (Fig. 1B). The miRNAs hsa-miR-421, hsa-miR-29b-1-5p, and hsa-miR-27b-5p were identified to be consistently upregulated in MKN74 cells with low expression of *CREBZF* (Fig. 1C). Then, we further studied two higher expressed miRNAs (hsa-miR-421, hsa-miR-29b-1-5p) of them and CREBZF in MKN74 cells and dysplasia tissues.

### Validation of hsa-miR-421 and hsa-miR-29b-1-5p expression in normal, low-grade, high-grade, and early GC dysplasia of patients

**Figure 2:**
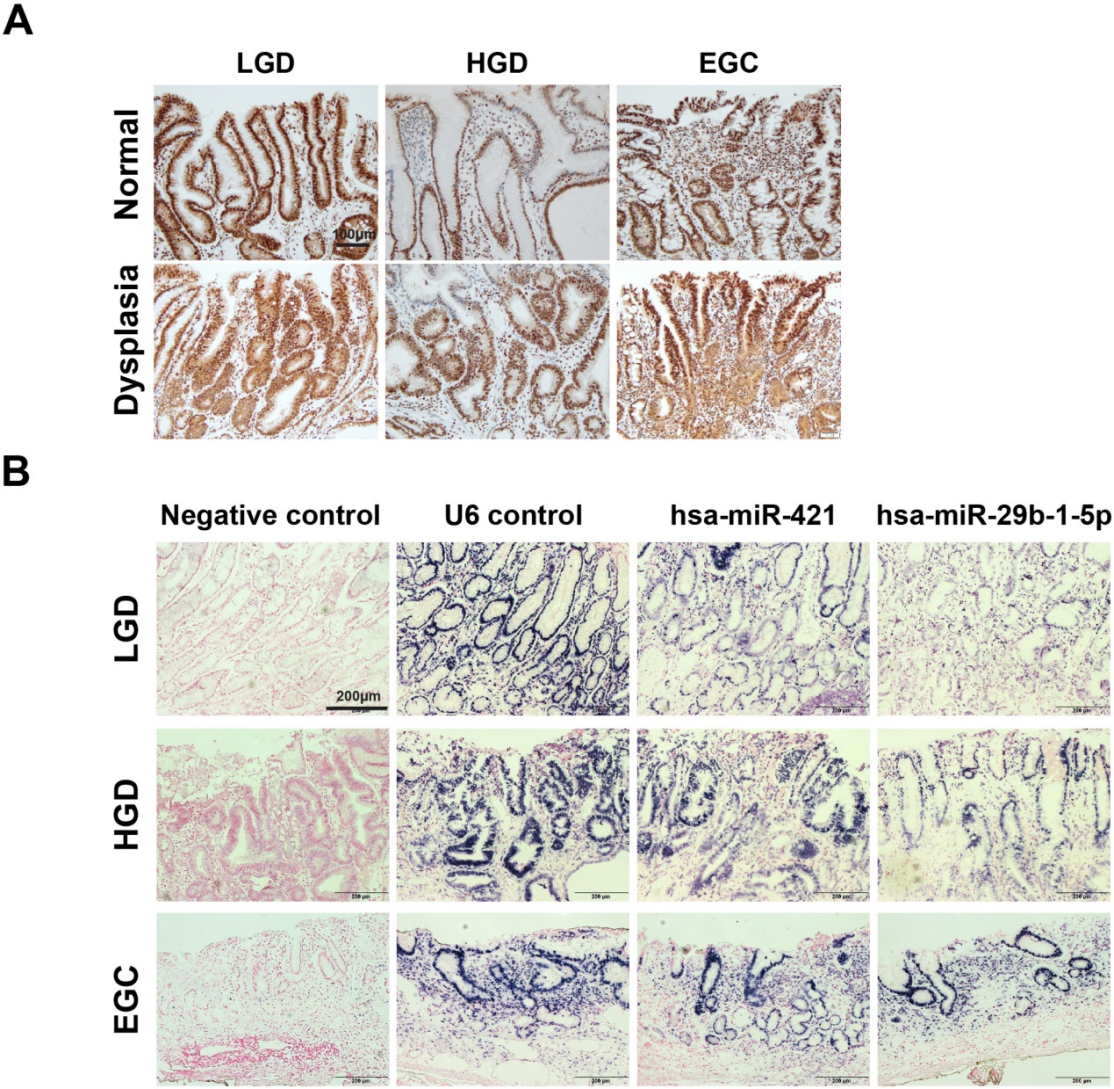
** Differential changes of CREBZF and miRNA expression in gastrointestinal biopsy tissues from low/high-grade dysplasia and early gastric cancer (EGC) patients.** (A) Representative immunohistochemistry stains of CREBZF between sample-matched normal (upper panels) and adenoma/dysplasia (down panels) of gastrointestinal biopsy tissues. Scale bar = 100 μm. (B) *In situ* hybridization of miRNAs (hsa-miR-421 and hsa-miR-29b-1-5p). *In situ* hybridization analyses using DIG-labeled miRCURY LNA microRNA detection probe complementary to hsa-miR-421 and hsa-miR-29b-1-5p were performed on paraffin sections of the gastrointestinal biopsy tissues. Scale bar = 200 μm. LGD, low-grade dysplasia; HGD, high-grade dysplasia; EGC, early gastric cancer.

Expression of CREBZF was detected in low/high-grade dysplasia tissues compared to normal pair tissues. Consistent with our previous findings (microarray and qPCR), the expression of CREBZF was found to be lower in invasive neoplastic glands of adenocarcinoma tissues than in normal pair tissues by immunohistochemical staining (Fig. 2A). Moreover, we investigated the expression of hsa-miR-421 and hsa-miR-29b-1-5p expression in different grades of adenoma dysplasia using *in situ* hybridization. The frequency and extent of hsa-miR-421 and hsa-miR-29b-1-5p expression showed a gradual increase with histologic progression from low, high, and early GC dysplasia of patients (Fig. 2B).

### miRNA (hsa-miR-421 and hsa-miR-29b-1-5p) can target CREBZF and regulate its expression

**Figure 3:**
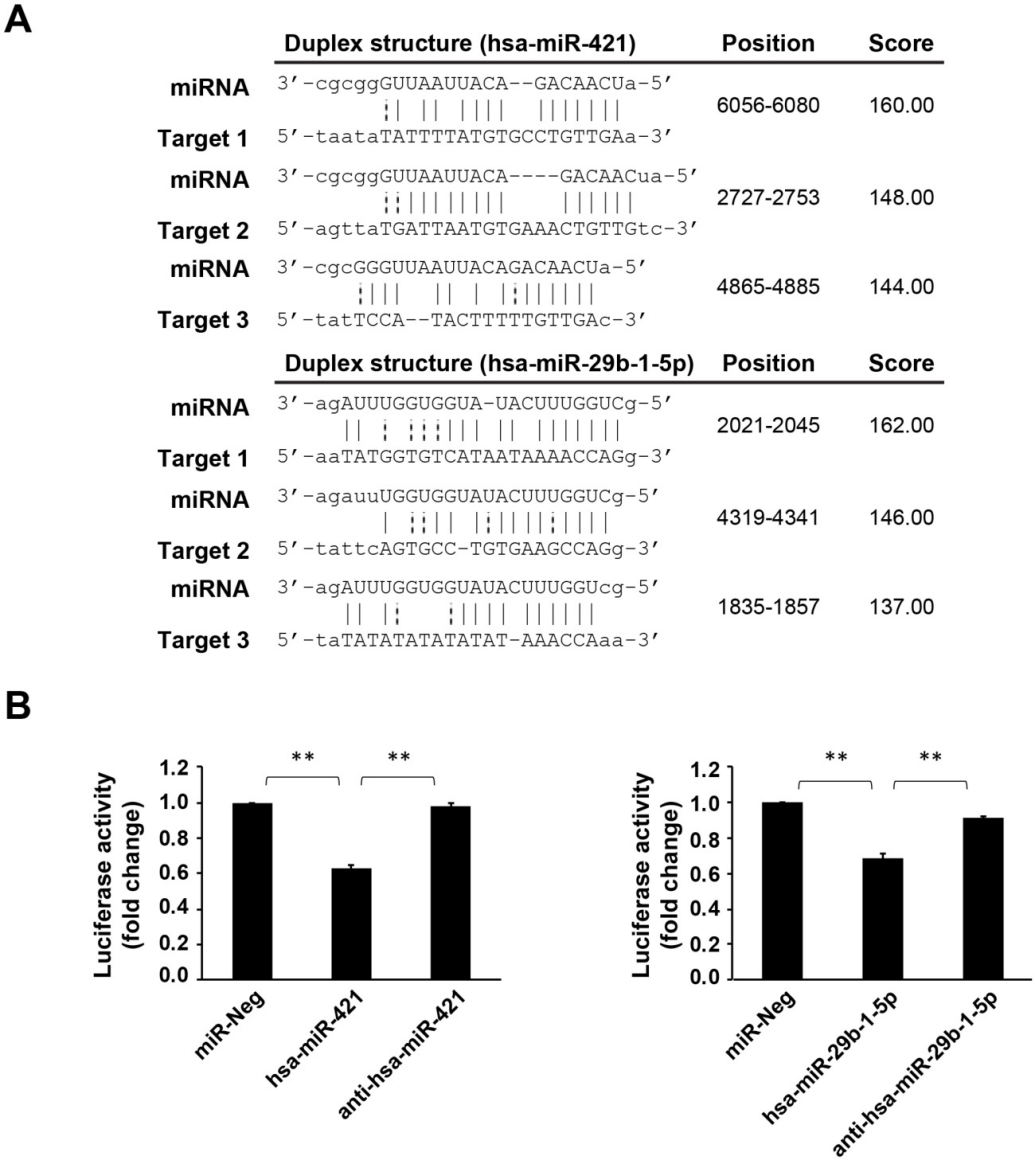
** The 3′UTRs of CREBZF contains the hsa-miR-421/hsa-miR-29b-1-5p binding site.** (A) Illustration of the hybridization between miRNA and the CREBZF 3′UTR binding site. miRNA-target interactions (Predicted by miRanda). (B) Luciferase assay using the 3′UTRs of CREBZF. miR-Neg: negative control miRNA. The data are presented as the mean ± STD of three separate experiments. (*p<0.05, **p<0.01)

**Figure 4:**
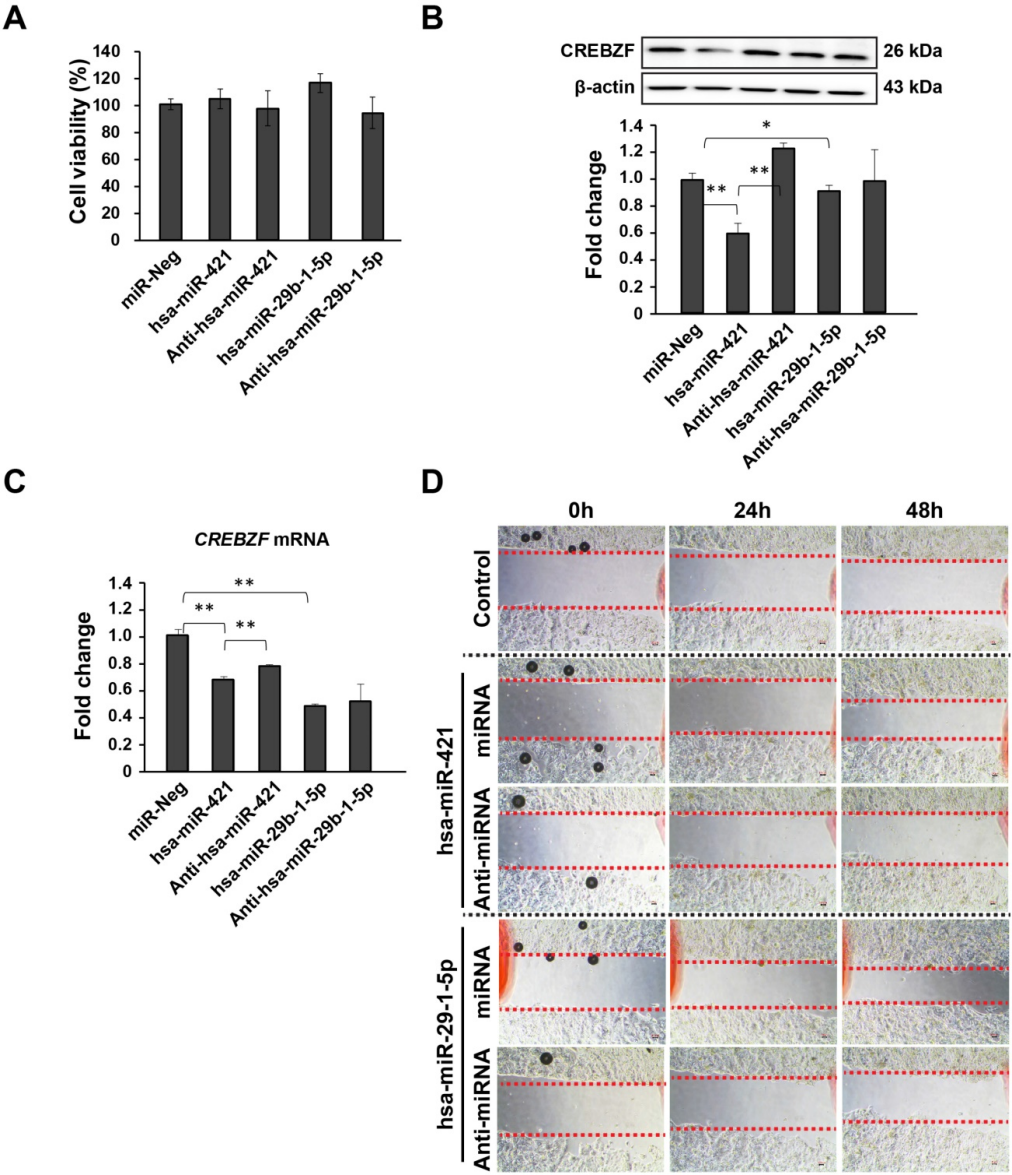
** Effects of CREBZF expression in MKN-74 gastric adenocarcinoma cells due to the activity of hsa-miR-421/hsa-miR-29b-1-5p.** (A) CCK-8 assays indicated that CREBZF inhibition by miRNA overexpression did not affect the proliferation of MKN-74 cells. (B) Representative images of western blot analysis. (C) Relative expression levels of CREBZF, and (D) migration. The data are presented as the mean ± STD of three separate experiments. (*p<0.05, **p<0.01)

Using bioinformatics databases, we confirmed that *CREBZF* is a target of these two miRNAs (hsa-miR-421 and hsa-miR-29b-1-5p) (Fig. 3A). As per the dual luciferase reporter assay, both hsa-miR-421 and hsa-miR-29b-1-5p could significantly inhibit the transcriptional activity of *CREBZF* but had no effect with negative control miRNA transfection (Fig. 3B). These data indicate that both hsa-miR-421 and hsa-miR-29b-1-5p target the 3′UTR regions of *CREBZF* mRNA in a sequence-specific manner. As depicted in Figure 4, hsa-miR-421 and hsa-miR-29b-1-5p possibly promote the proliferation and migration/invasion of GC cells through inhibition of *CREBZF* expression.

### miRNA (hsa-miR-421 and hsa-miR-29b-1-5p) promote the migration of MKN-74 cells but not proliferation

To explore the possible role of miRNA (hsa-miR-421 and hsa-miR-29b-1-5p) on GC, MKN-74 cells transfected with miRNA mimics and anti-miRNAs showed improvement and reduction in cell migration but not proliferation (Fig. 4). CCK-8 assays revealed that miRNA transfection does not affect the proliferation of MKN-74 cells (Fig. 4A). Then, we further determined how these two miRNAs affect *CREBZF* at both the mRNA and protein levels (Fig. 4B and 4C). Western blot analysis showed that miRNA mimic treatments could significantly decrease CREBZF expression levels in MKN-74 cells (Fig. 4B). Knockdown of miRNAs with anti-miRNAs again increased CREBZF expression in MKN-74 cells. qRT-PCR analysis also confirmed these changes at the mRNA level (Fig. 4C). Further experiments revealed that upregulating miRNA expression contributed to migration in MKN-74 cells by CREBZF down-expression (Fig. 4D). These data indicate that *CREBZF* has an inhibitory effect in the migration of MKN-74 cells by miRNA inhibition.

## Discussion

In this study, we showed that hsa-miR-421 and hsa-miR-29b-1-5p, acting as GC development enhancers, regulated GC cell migration by targeting *CREBZF*. The upregulation of these miRNAs was observed in gastric adenoma/dysplasia and GC cells in our results. Moreover, knockdown of miRNAs with anti-miRNAs decreased GC cell migration with increase in *CREBZF* expression in GC cells.

Irene et al. reported that CREBZF is as a positive regulator of p53 activity 11. They suggested that CREBZF, a member of the mammalian ATF/CREB family of transcription factors, may participate in the modulation of p53 tumor suppressor function. p53 is a key tumor suppressor, which is reflected by the fact that inactivation of the p53 pathway occurs in most human cancers. In line with the report, our results indicated that higher expression of CREBZF is related to the repression of GC. Moreover, why CREBZF is downregulated in gastric adenoma/dysplasia is an interesting phenomenon that requires further investigation. However, the expression levels and role of CREBZF remain unknown in GC development.

Increasing evidence has shown that miRNAs are closely related to GC progression. Because miRNAs can negatively regulate gene expression, we hypothesized that miRNAs may be involved in GC progression. For example, miR-107 and miR-25 simultaneously target LATS2 and regulate proliferation and invasion of GAC cells 12. In a recent study, miR-5590-3p directly targeted DDX5, and ectopic miR-5590-3p caused a reduction in DDX5 and activated the AKT/m-TOR pathway 13. DDX5-mediated phosphorylation of mTOR upregulates cyclin D1 to drive the proliferation of GC cells, which supported our data that miR-5590-3p inhibited cyclinD1 via the DDX5/m-TOR pathway in GC cells. miR-589, as an oncogene, markedly induced cell metastasis and invasion via an atypical miR-589-LIFR-PI3K/AKT-c-Jun feedback loop, which suggested that miR-589 is a potential biomarker and/or therapeutic target for GC management 14. miR-99b-5p/203a-3p and miR-99b-5p/203a-3p may function as tumor suppressive miRNAs by negatively regulating IGF-1R expression in GC cells 15. miR-4455 functions as a tumor suppressor in GC by targeting VASP, leading to the activation of the PI3K/AKT signaling pathway and inhibition of VASP-mediated proliferation, migration, and invasion of GC cells 16. miR-23a/27a/24-2 cluster may mediate the progression of GC through the suppression of SOCS6 expression 17.

miR-421 plays a key role in cancer progression and upregulation of miR-421 in plasma, occurring initially in precancerous gastric lesions as well as in the early stage of GC 18. Few published studies have addressed the potential association between microRNA-421 expression and prognosis of GC 19-21. Moreover, miR-421 levels in gastric juice from gastric patients were not significantly associated with the main clinicopathological factors such as tumor size, Lauren's classification, and Borrmann's classification 22. However, for the detection of early GC, the use of gastric juice miR-421 showed a remarkable improvement compared with that observed from use of serum carcinoembryonic antigen alone.

In our previous study, we suggested that three miRNAs (hsa-miR-421, hsa-miR-29b-1-5p, and hsa-miR-27b-5p) in conjunction with two mRNAs (FBXO11 and CREBZF) might play an important role in the development of GC from premalignant adenomas. We further investigated the role of CREBZF in GC cells via cell viability and invasion assays by miRNA/anti-miRNA modulation in MKN-74 GC cells. However, we did not carry out a large-scale study of gastric adenoma/dysplasia and adenoma cell lines to identify miRNAs with CREBZF involved in GC progression, which remains one of the limitations of this study. Further studies using more patient groups and adenoma cell lines will be helpful to identify potential miRNAs involved in GC progression.

Our data showed that CREBZF is a target gene of hsa-miR-421/hsa-miR-29b-1-5p and might play an important role in the development of GC from gastric adenoma/dysplasia. This is the first study to identify CREBZF as a key repressor and that it can be negatively regulated by hsa-miR-421 and hsa-miR-29b-1-5p to promote GC progression. Our findings provide new insights into the biological functions of CREBZF with miRNAs and the molecular mechanisms of GC progression.

## Figures and Tables

**Table 1:** Clinicopathological features of 12 patients.

Patient No.	Gender	Age	Histologic diagnosis	Helicobacter pylori status
1	M	73	Low-grade dysplasia	Positive
2	M	59	Low-grade dysplasia	Negative
3	F	76	Low-grade dysplasia	Positive
4	M	48	High-grade dysplasia	Positive
5	M	59	High-grade dysplasia	Positive
6	M	66	High-grade dysplasia	Negative
7	M	70	Early gastric cancer	Positive
8	M	71	Early gastric cancer	Positive
9	M	64	Early gastric cancer	Positive
10	M	69	Early gastric cancer	Negative
11	M	83	Early gastric cancer	Positive
12	M	73	Early gastric cancer	Positive

**Table 2:** Sequences of primers used for real-time RT-PCRs.

Genes		Primer sequence (5ʹ - 3ʹ)
*CREBZF*	F	TGTGCCTGTTGAAAGAACAAATC
	R	ACATAAAGCTGTGCTGCCAAA
*FBXO11*	F	TTGCCGAAAAGAACAGCGTG
	R	AGCAGGTGCTGCTGATAGAT
*Internal control*		
*GAPDH*	F	GAAAGCCTGCCGGTGACTAA
	R	AGGAAAAGCATCACCCGGAG

a) F, sequence from sense strands; b) R, sequence from antisense strands.
